# Neuropsychological Profiles Correlated with Clinical and Behavioral Impairments in a Sample of Brazilian Children with Attention-Deficit Hyperactivity Disorder

**DOI:** 10.3389/fpsyt.2015.00163

**Published:** 2015-11-26

**Authors:** Sueli Rizzutti, Viviane Schuch, Bruno Muszkat Augusto, Caio Colturato Coimbra, João Pedro Cabrera Pereira, Orlando Francisco Amodeo Bueno

**Affiliations:** ^1^Psychobiology Department, Universidade Federal de São Paulo, São Paulo, Brazil; ^2^Universidade Santo Amaro, São Paulo, Brazil

**Keywords:** comorbidities, ADHD, CBCL, DESR-CBCL, neuropsychological profile

## Abstract

Attention-deficit hyperactivity disorder (ADHD) is a complex neurodevelopmental disorder that implies several-step process, and there is no single test to diagnose both ADHD and associated comorbidities, such as oppositional-defiant disorder (ODD), anxiety disorder, depression, and certain types of learning disabilities. The purpose of the present study was to examine correlations between behavioral and clinical symptoms by administering an extensive neuropsychological battery to a sample of children and adolescents from a developing country. The sample was divided into three groups: non-ADHD, ADHD-non-comorbid, and ADHD + comorbidity. A full neuropsychological battery and clinical assessment found that 105 children met DSM-5 criteria, of whom 46.6% had the predominantly inattentive presentation, 37.3% had combined presentation, and 16% were predominantly hyperactive/impulsive presentation. The internal correlation between neuropsychological tests did not reach statistical significance in the comparison between ADHD and non-ADHD cases (*p* < 0.17). Clinical ADHD cases, including both + comorbidity and non-comorbid groups, performed substantially worse on continuous performance test (CPT), working memory. Comparing ADHD-non-comorbid and ADHD + comorbidity groups, the latter did significantly worse on inhibitory control, time processing, and the level of perseveration response on CPT indexes, as well as on working memory performance and child behavior checklist (CBCL) tests particularly the CBCL-deficient emotional self-regulation test in the ADHD + comorbidity group. Children diagnosed as ODD or with conduct disorder showed close correlations between clinical CBCL profiles and externalized symptoms. Our findings suggest that ADHD + comorbidity and ADHD non-comorbid cases may be differentiated by a number of neuropsychological measures, such as processing speed, inhibitory control, and working memory, that may reflect different levels of involvement of the hot and cool executive domains, which are more impaired in cases of severe symptomatic-externalized behavior and emotional regulation problems. Therefore, profiles based on clinical and behavioral findings can help clinicians select better strategies for detecting neuropsychological impairment in Brazilian children with ADHD.

## Introduction

Attention-deficit hyperactivity disorder (ADHD) affects approximately 5.9 and 7.1% of children and adolescents across different cultures and are characterized by persistent pattern of inattention, hyperactivity, and/or impulsivity (1, 2). The lack of a gold-standard protocol for clinical and neuropsychological assessment of ADHD reinforces the need for studies that establish objective multiple variables and help decide which tools will be most appropriate for the diagnosis of ADHD.

Several studies showed a high prevalence of comorbidities of approximately 20–50% (3, 4), and children with ADHD tend to present learning difficulties, behavioral and conduct disorders (CDs), mood and motor problems, and delayed development of speech (5). Associations with comorbidities, such as oppositional-defiant disorder (ODD), anxiety disorder (AD), depression disorder (DD), and learning disabilities (LD) require a more comprehensive multidisciplinary assessment. Many researchers believe that the diagnoses based on the DSM-IV are over-diagnosing because many children would be excluded by broadening the scope of assessment through detailed neuropsychological testing to delimit the areas involved, such as selective, alternating, and sustained attention, in addition to common executive dysfunctions in ADHD (6). Many studies have recommended a neuropsychological evaluation for diagnostic and complementary determination of ADHD cases (7). Sustained attention tests, such as the continuous performance test (CPT) (8, 9) in addition to a broad spectrum of cognitive tests, scales, and questionnaires, have been used to determine specific deficits with emphasis on predominant disorders in associative areas not only related to attention but also related to executive functions (EFs), such as functional memory and cognitive flexibility. Consequently, Conners’ CPT provides 15 measurements that potentially reflect different dimensions of attention. Furthermore, few studies applying Conners’ CPT have used the full potential of the test to analyze several dimensions of attention (9).

Convergent data from various sources, including neuroimaging, neuropsychological, genetics, and neurochemical studies, have generally implicated fronto-striatal network abnormalities as contributing to ADHD (10, 11). Studies have shown regional abnormalities including lower volume of the basal ganglia (putamen, globus pallidus, and caudate nucleus) in ADHD (11, 12), which is in accordance with the fronto-striatal models pathophysiology of this disorder (13). Nakao et al. (14) also identified a reduction in the overall volume of gray substance in the group with ADHD.

Abnormal brain networks can be related to this additive substrates of the observed synergistic dysfunctional neuropsychological performance present in the ADHD+ cases. Left dorsolateral prefrontal cortex (DLPFC) and anterior cingulated cortex (ACC) have been associated with interference control assessment, work memory, and EF (15). It is well established that DLPFC and ACC are key impaired brain areas in ADHD (16). The most replicated alterations in childhood include significantly smaller volumes in the DLPFC, caudade, pallidum, corpus callosum, and cerebellum (17).

A child behavior checklist (CBCL) (18) profile has been identified that characterizes children with significant clinical symptoms of aggression, attention/hyperactivity problems, and anxiety/depression (19, 20). Moreover, cross-sectional and short-term follow-up studies have shown that a CBCL profile is stable throughout childhood (21) and is associated with increased risk of cognitive problems (11, 22) and severe disruptive behavior disorder (23–26). The CBCL is a broad spectrum inventory that records, in a standardized format, the behavioral and emotional problems and competencies of children aged 4–18, as reported by their parents or parent surrogates. It is scored on social competence and behavior problem scales (25).

Child behavior checklist scores may provide useful information for differential diagnosis when used in combination with other clinical data (e.g., background information, clinical observations, and other assessment data) (27). Biederman et al. (28) suggested that poor emotional self-regulation relates to difficulty in the regulation of physiological arousal in many situations, such as the presence of strong emotions, difficulty inhibiting inappropriate behaviors in response to positive or negative emotions, difficulty refocusing attention due to strong emotions, and disorganized behavior in response to emotional activation.

Biederman et al. (28) and Spencer et al. (29) sought to better discriminate between different levels of difficulty severity in relation to poor emotional self-regulation among ADHD children and adolescents. For this purpose, they used CBCL scales for anxiety/depression, aggression, and attention problems, they defined deficient emotional self-regulation (DESR) if a child had an aggregate cut-off score of >180 but <210 on the anxiety/depression, aggression, and attention scales of the CBCL (CBCL-DESR). This criterion was chosen due to congruence with the clinical concept of DESR, and because the high end (>210) had previously been associated with severe mood and behavioral dysregulation in ADHD children.

Other behavioral rating scales may also be useful to assess ADHD children in ecological contexts (family and school), including the Conners’ rating scaling (30), the ADHP rating scale (31), and the attention-deficit disorders’ evaluation scale (ADDES) (32). However, the issue of which tools are most appropriate and sensitive for the clinical heterogeneity of ADHD remains controversial, as is the delimitation of the clinical and neuropsychological indicators used to assess children and adolescents with suspected ADHD. Few studies in children suggest that ADHD with comorbidities might promote synergistic neuropsychological deficits when compared to ADHD only, especially in processing and naming speed, working memory, and response inhibition (33, 34). In fact, few studies conducted a direct comparison between children with ADHD alone or comorbid including differences in CBCL-DESR in developing countries (28, 29).

Thus, our primary objective was to examine correlations between behavioral and clinical symptoms by administering an extensive neuropsychological battery to a sample of ADHD children and adolescents with and without comorbidities in a sample of Brazilian children.

## Materials and Methods

Ethics committee approval was obtained as was all parents’ informed consent before enrollment.

One hundred and twenty-four children aged 6–14 were selected through voluntary enrollment by parents attending the ADHD Outpatient Sector at NANI-UNIFESP following complaints of restlessness, attention difficulties, and/or impulsivity. They were then asked to attend an individual trial interview. Children who met the inclusion (at least 6 DSM-5) for hyperactivity and/or 6 for inattention (starting before the age of 12) were referred to a multidisciplinary assessment schedule, which consisted of medical and neuropsychological evaluations, as well as social, neurological, and psychiatric examinations and family assessment. All the children were not taking medication in the period of evaluation.

The neuropsychological battery included:

(1) Intellectual level: Wechsler Intelligence Scale for Children abbreviated WISC-III (35).
(2) EACIP scale (36): teachers assessed five main areas of child behavior: hyperactivity/conduct problems (EACIP-I), independent functioning (EACIP-II), inattention (EACIP-III), neuroticism/anxiety (EACIP-IV), and social interaction (EACIP-V).
(3) Conners’ CPT (37): computerized test of sustained attention and mental flexibility.
(4) Working memory (38): (a) forward and backward digit span assesses the phonological loop component of working memory and (b) forward and backward Corsi blocks assesses the ability to reproduce a sequence of stimuli (blocks) in a visual–spatial design assessing the sketchpad component of working memory.
(5) Rey figure: assesses visual-constructive functions (Rey copy) as well as visual memory (Rey memory) when subjects draw a complex figure from memory (39).
(6) The widely used CBCL, which takes parent-reported data for 112 behavioral problems and three areas of competency (40), has been translated into Portuguese and validated for Brazilian children (41). Parents (usually mothers) or guardians were asked to complete the CBCL as a part of the evaluation process before the final clinical diagnoses. DESR was characterized when a child had an aggregate cut-off score of >180 but <210 on the anxiety/depression, aggression, and attention scales of the CBCL (CBCL-DESR ADHD) (42).

Using our interdisciplinary criteria, the ADHD diagnosis was based on DSM-5 and neurologic and psychiatric evaluations. The diagnosis of psychiatric comorbidities was based on the CBCL and one structured psychiatric interview. In relation to comorbidities, the assessment was conducted by two board-certified doctors (a psychiatrist and a neuropediatrician) with great experience (over 25 years) in clinical evaluation and research. Data were collected over 2 days of testing. At the end of this evaluation, their cases were discussed by the service team, and the diagnoses were established. All diagnostic uncertainties were resolved by excluding cases in which cognitive and clinical data were non-congruent. Children presenting symptoms of inattention, hyperactive-impulsivity, and ADHD combined with comorbidities (ADHD + comorbidity) or without comorbidities (ADHD-non-comorbid) were included in the study.

### Statistical Analysis

Clinical and neuropsychological test scores were compared using multivariate analysis of variance, followed by Tukey’s test and Pearson’s correlation coefficient to assess correlations between clinical and neuropsychological variables. Three groups were compared: non-ADHD, ADHD-non-comorbid, and ADHD + comorbidity. All tests were two tailed, and alpha was set at 0.05.

## Results

Most children were males (70; 66.6%). The average age was 7 years and 7 months and ranged from 6 to 12 (SD 1.6). In relation to DSM-5 ADHD, 105 (68.1%) children met at least 6 criteria for inattentiveness and/or hyperactivity; 49 cases (46.6%) were predominantly inattentive presentation; 39 (37.3%) showed hyperactivity and inattention symptoms combined (combined presentation); and 17 (16%) showed predominantly hyperactivity plus impulsivity presentation. Of the remainder of the 154 children, those not meeting ADHD criteria were 49 (31.9%), of whom seven presented learning disabilities (14.3%), 10 (20.4%) had altered family dynamics, 16 (32.6%) had adaptive disorders, and 16 (32,6%) had a mental deficit (QI < 85) (see Table 1). Of the 105 children with DSM-5 ADHD for inattentiveness and/or hyperactivity, 46 (44%) presented comorbidities, 30 (66.6%) presented AD, 11 (24.2%) had ODD, and 3 (9%) had CD. With regards to mood disorders, the study found four (9%) cases of bipolar disorder (BD), and four (9%) met the criteria for severe DD (Table 1).

**Table 1:** **Clinical diagnosis and gender distribution**.

Clinical characteristics	*n* (%)	Gender	
		M	F
**ADHD**	105 (68.1)	70	35
ADHD – inattentive mode of clinical presentation	49 (46.6)	24	25
ADHD – combined mode of clinical presentation	39 (37.3)	32	7
Hyperactivity/impulsivity predominance	17 (16)	14	3
**DESR + (ADHD with comorbidity)**	46 (44)	33	13
**No ADHD**	49 (31.9)	35	14
Learning disabilities	7 (14.3)	4	3
Alterations in family dynamics	10 (20.4)	6	4
Mental disability (<85)	16 (32.5)	9	7
Adaptive difficulties	16 (32.6)	20	6
**ADHD with comorbidity**	59 (56)	33	26
**ADHD without comorbidity**	46 (44)	27	19
Anxiety disorder	30 (66.6)	19	11
Oppositional-defiant disorder	11 (24.2)	9	2
Conduct disorder	4 (9)	3	1
Bipolar disorder	4 (9)	3	1
Depression	4 (9)	3	1

*M, male; F, female*.

In terms of global cognitive performance, the average IQ was 100 (range from 85 to 126).

In relation to performance on the CPT, ADHD children had more difficulty with omission, commission, reaction time, variability, and perseverance than non-ADHD children. However, the number of omissions was significantly higher for children from the inattentive group than the combined group, which suggests the omission measure may be a differential criterion. In fact, 44% of ADHD+ comorbidity children met the criteria for DESR in ODD (11 patients), CD (3 patients), BD (4 patients), and AD (28 patients).

Table 2 shows the correlations for clinical symptoms of inattentiveness (>6 DSM-5), hyperactivity, and impulsivity (>6 DSM-5), who were the only ones presenting a significant correlation for omission, Hit RT (SE), Hit RT block change, and variability score. Children with impulsivity presented lower variability in response style (−0.54). No difference was found for other CPT measures in the group of predominantly inattentive or hyperactive children.

**Table 2:** **Distribution of mean scores on CPT and CBCL**.

	CBCL oppositional	CBCL conduct	CBCL attention	CBCL time
CPT omission	−0.015	0.105	0.139	0.078
	0.924	0.514	0.387	0.627
	41	41	41	41
CPT commission	−0.038	−0.047	0.044	−0.101
	0.814	0.773	0.786	0.529
	41	41	41	41
CPT Hit Rt	0.130	0.176	0.109	0.022
	0.419	0.271	0.499	0.893
	41	41	41	41
CPT perseveration	−0.130	0.051	0.102	0.055
	0.419	0.753	0.524	0.734
	41	41	41	41

Table 3 shows correlations between CPT measures and neuropsychological tests for children with ADHD (combined and inattentive). A negative correlation (−0.64) was found between forward and backward digit spans (phonological working memory) and omission scores (−0.65, −0.63). There was also a negative correlation between reaction time (Hit RT and Hit RT SE) scores and forward digit span (−0.64) and backward visual-spatial working memory (Corsi test). Children with high hyperactivity and impulsivity scores on behavioral scales showed poor performance on working memory tasks.

**Table 3:** **Distribution of mean scores on neuropsychological tests**.

Neuropsychological assessment	No ADHD (*n* = 49) group 1 (mean + SD)	ADHD (*n* = 39) group 2 (combined) (mean + SD)	ADHD (*n* = 49) group 3 (inattentive) (mean + SD)	Statistic test
EACIP – hyperactivity	60.8 (22)	82.8 (28.3)	57.9 (45.8)	*F*_(112, 2)_^a^,^b^,^c^=
EACIP – inattentiveness	14.6 (85)	15.6 (7.6)	15.0 (8.6)	*F*_(112, 2)_=
EACIP – negative social interaction	9.2 (5.4)	8.2 (5.4)	4.3 (4.5)	*F*_(112, 2)_=
Rey Fig copy	60 (11)	32.3 (12)	44.6 (11)	*F*_(112, 2)_^a^,^b^,^c^=
Rey Fig memory	80.8 (13)	51.8 (13)	40.6 (8)	*F*_(112, 2)_^a^,^b^,^c^=
Digits (backward)	4 (1.3)	2.6 (1.7)	2.1 (1.0)	*F*_(112, 2)_=
Digits (forward)	5 (0.6)	4 (0.6)	3.8 (0.7)	*F*_(112, 2)_=
Corsi (forward)	4.3 (0.6)	4.4 (0.7)	4.1 (0.6)	*F*_(112, 2)_=
Corsi (backward)	4.0 (1.1)	3.0 (1.2)	2.3 (1.2)	*F*_(112, 2)_=
CPT omission	12.2 (12.7)	30.2 (28.7)	52.9 (34.2)	*F*_(112, 2)_^a^,^b^,^c^=
CPT commission	12.3 (8)	21.5 (8)	25.3 (5.2)	*F*_(112,2)_^a^,^b^=
Reaction time	337.3 (80)	537.3 (100)	641.1 (90.3)	*F*_(112,2)_^a^,^b^=
Variability	21.5 (20)	41.5 (22)	58.1 (13.3)	*F*_(112, 2)_^a^,^b^=
Perseveration	11.1 (12)	20.1 (12)	19.3 (12.6)	*F*_(112, 2)_^a^,^b^=

*^a^Significant difference between groups 1 and 2*.

*^b^Significant difference between groups 1 and 3*.

*^c^Significant difference between groups 2 and 3*.

## Discussion

This study aimed to compare the neuropsychological profile of symptomatic children and adolescents presenting comorbidity with ADHD with those suffering from ADHD without comorbidity.

In recent years, the role of multidisciplinary evaluation of ADHD has been constantly reviewed and involved several centers in different countries (28). Therefore, the current priority is to find new ways of making the diagnosis more precise. ADHD comorbidities, such as ODD, AD, DD, and LD, can be more accurately diagnosed after multidisciplinary and neuropsychological assessment. Using a detailed testing protocol, our study found that only 31.9% of cases did not meet broader clinical criteria (Table 1). This finding emphasizes the risk of ADHD diagnosis being based on behavioral scales that are not completely precise instruments for the detection of neuropsychological endophenotypes, including teacher-reported behavioral scales. In this study, the hyperactivity variable measured by the EACIP scale was the only one to show a positive correlation with the final ADHD diagnosis (Table 3). Many different hypotheses relating to the neuropsychological impact on ADHD have prompted several studies and efforts to determine the clinical advantages of neuropsychological tests in multiple areas related to motor inhibition (37), planning and organization (39), and working memory (38).

Conners’ CPT is widely utilized to test for sustained attention and inhibition due to ease of administration and a broad profile allowing age-group distribution for parameters closely related to attention ability, such as reaction time, omission, and commission errors.

Our study showed that Conners’ CPT was sufficiently sensitive to distinguish cases that met the criteria for clinical ADHD from non-ADHD profiles, but not for absolute differentiation between the ADHD modes of clinical presentation, because significant differences were found for omission scores alone (Table 2). ADHD + comorbidity children presented slower reactions and more omission and commission errors than ADHD-non-comorbid or non-ADHD children, which matches findings reported in the literature (43, 44). The specificity of the CPT variables is quite contradictory and sometimes inconsistent, especially when differentiating the ADHD mode of clinical presentation (45, 46). Furthermore, the small differences found in the correlations between the ADHD mode of clinical presentation may have been influenced by the relatively small number of cases in the hyperactive/impulsive clinical presentation, but this finding is in line with those of other authors (28), who reported that although the CPT is highly sensitive (approximately 88% when detecting ADHD), it not very specific (20–37%) for different modes of clinical presentation. On the other hand, in our study, ADHD cases that met the criteria for oppositional and/or CD presented a higher frequency of omission, reaction time, Hit RT block change and variability indices (Table 2).

Conners’ CPT as an isolated paradigm that is not ideal for diagnosing the modes of clinical presentation, but it is useful for assessing the overall impact of ADHD on the cognitive domain and for separating purely contextual cases. It is also particularly sensitive for cases of comorbidities, such as ODD and CD (Table 2).

For Rey complex figure visual-constructive and visual-spatial skills, we found a negative correlation between percentile perseveration in Conners’ CPT and drawing a figure from memory. The higher the performance on the memory test, the lower the perseveration on the CPT (Table 3). We contend that this result is related to the EF in ADHD children. Homack and Riccio (47) and Shin et al. (48) suggested that while the Rey complex figure is very suitable for delimiting patterns of spatial planning/organization, it is not sufficiently specific to differentiate ADHD children. The Rey complex figure, which requires spatial planning and visual-constructive skills, proved to be a useful means of assessing the effect of attention on spatial functions. In this respect, its figure copy and memory modules may reflect the modulation of visual-selective attention and self-monitoring, which are essential for organizing and planning complex activities (39), thus suggesting that poor performance on this task associated with higher scores for perseveration on the CPT may reflect an executive dysfunction.

The negative correlations (Table 3) we observed between working memory scores (digits span and Corsi blocks tests) and omissions and commissions on the Conners’ CPT reinforced the key role of functional memory as the origin of cognitive and behavioral functions associated with ADHD (1, 47).

In combined ADHD cases (score >6 for hyperactivity), performance correlated positively with more omissions, reaction time, and variability of answers in the CPT (Table 2).

Several studies showed a high prevalence of comorbidities of approximately 20–50% (4, 5). Our study found comorbidities in 44% of diagnosed cases, which shows the importance of an interdisciplinary approach, because many disorders associated with ADHD, such as AD and CD, require different clinical interventions and approaches. We also found that ADHD + comorbidity children with ODD and CD performed worse on the CPT (Table 2). This reinforces the idea that comorbidities have a greater impact on cognitive, executive, and attention performance of ADHD patients (49). The impairment in brain circuits most replicated in both ADHD (50) and comorbidities (51) is related to the frontal and DLPFC being linked to inhibitory control tasks more pronounced in comorbid cases. Thus, impaired interference control observed in CPT, such as commission and omission scores, might be a potential neuropsychological signature of clinical severity or suggest involvement of additional limbic areas such as orbitofrontal cortex modulating emotional regulation process (34).

Although ADHD has long been conceptualized as a deficient self-regulation disorder, researchers have only recently focused on DESR. DESR refers to (1) deficits in self-regulation of physiological arousal due to strong emotions, (2) difficulties inhibiting inappropriate behavior in response to either positive or negative emotions, (3) problems refocusing attention from strong emotions, and (4) disorganization of coordinated behavior in response to emotional activation (20). Clinically, DESR traits include low frustration tolerance, impatience, being quick to anger, and being easily excited to react emotionally. Although these traits may be a source of significant morbidity, there has been little research on the subject and uncertainties remain as to how best to measure it. We found that ADHD children via CBCL-DESR profile were at a significantly increased risk for social impairment, including poorer functioning with peers, siblings, and parents in all four major role areas: school, spare time, peer relations, and home life. These findings are consistent with those of Whalen and Henker (52) in that emotional dysregulation in ADHD children disrupted reciprocity and cooperative activities involved in peer relationships, as well as the findings by Barkley (53) in that DESR might account for a significant portion of the prominent social dysfunction documented in ADHD. The latter speculated that DESR may trigger negative reactions from peers, siblings, and parents which in turn leads to further poor social responses from the affected child (53). Some children fail to achieve behavioral and attentional self-regulations as they grow up leading to disruptions in their adaptive and academic functioning in school, daycare, and at home (54).

ROC curve analyses showed close correlations with CBCL scales for ADHD + comorbidity children, particularly ODD and CD cases via Pearson’s test (0, 61). ODD cases showed CPT score alterations especially for vigilance adaptation (Hit RT), commission, and perseveration. These findings may be related to low inhibitory and impulsivity control in ADHD + comorbidity cases.

Although our paper only reassures the known close relation between the severity of neuropsychological profile with behavioral symptoms of children with ADHD (8, 23, 42), it brings complementary data suggesting that in cases with comorbidities there is also a greater involvement of large neural networks. The distinction between cool (cognitive modulation of information) and hot EFs linked to emotional self-regulation and the reward system might underlying such different traits. It can be essential to identify behavior risk factors in these children. Therefore, early recognition of such emotional markers might help the multidisciplinary team to recognize neuropsychological patterns to adopt preventive measures including psychological support, parental guidance, and appropriate drug treatment.

Overall, our study suggests multiple connections between neuropsychological test scores, which are useful for a more detailed delimitation of the clinical profile and outcome signaling comorbidities in ADHD children. Therefore, our study provides evidence regarding the neuropsychological deficits of these patients, so that techniques that might improve low inhibitory and interference control should be included in the rehabilitation techniques in children and adolescents with ADHD + comorbidities. A larger sample will help us to determine clinical and neuropsychological indicators that may lead not only to a more precise diagnosis but early detection of neurocognitive outcome that can also help us to select rehabilitation strategies for better social and cognitive inclusion of ADHD children and adolescents.

### Limitations

This study should be considered in the light of its limitations. Data were derived from a limited sample, and as such, our results might not be extrapolated to clinical settings. Co-occurrence of internalized and externalized problems and demographic and clinical heterogeneity of the samples may limit generalization of our findings.

## Conflict of Interest Statement

The authors declare that the research was conducted in the absence of any commercial or financial relationships that could be construed as a potential conflict of interest.

## References

[1] American Psychiatric Association. Diagnostic and Statistical Manual of Mental Disorders. 5th ed Washington, DC: American Psychiatric Association (2013). 10.1176/appi.books.9780890425596

[2] Willcutt EG. The prevalence of DSM-IV attention-deficit/hyperactivity disorder: a meta-analytic review. Neurotherapeutics (2012) 9(3):490–9. 10.1007/s13311-012-0135-8 22976615 PMC3441936

[3] Polanczyk G Lima MS Horta BL Biederman J Rohde LA The worldwide prevalence of ADHD: a systematic review and meta-regression analysis. Am J Psychiatry (2007) 164:942–8. 10.1176/ajp.2007.164.6.942 17541055

[4] Tramontina S Schmitz M Polanczyk G Rohde LA. Juvenile bipolar disorder in Brazil: clinical and treatment findings. Biol Psychiatry (2003) 53:1043–9. 10.1016/S0006-3223(03)00008-8 12788249

[5] Kooij SJ Bejerot S Blackwell A Caci H Casas-Brugué M Carpentier PJ European consensus statement on diagnosis and treatment of adult ADHD The European Network Adult ADHD. BMC Psychiatry (2010) 10:67. 10.1186/1471-244X-10-67 20815868 PMC2942810

[6] Bush G. Attention-deficit/hyperactivity disorder and attention networks. Neuropsychopharmacology (2010) 35(1):278–300. 10.1038/npp.2009.120 19759528 PMC3055423

[7] Palumbo DR Diehl J Managing attentional disorders. In: Hunter SJ Donders J, editors. Pediatric Neuropsychological Intervention. Cambridge: Cambridge University Press (2007). p. 253–86.

[8] Solanto MV Gilbert SN Raj A Zhu J Pope-Boyd S Stepak B Neurocognitive functioning in AD/HD, predominantly inattentive and combined subtypes. J Abnorm Child Psychol (2007) 35(5):729–44. 10.1007/s10802-007-9123-6 17629724 PMC2265203

[9] Epstein JN Erkanli A Conners CK. Relations between continuous performance test performance measures and ADHD behaviors. J Abnorm Child Psychol (2003) 31:543–54. 10.1023/A:1025405216339 14561061

[10] Schneider M Retz W Coogan A Thome J Rosler M Anatomical and functional brain imaging in adult attention-deficit/hyperactivity disorder (ADHD) – a neurological view. Eur Arch Psychiatry Clin Neurosci (2006) 256(Suppl 1):i32–41. 10.1007/s00406-006-1005-3 16977550

[11] Vaidya CJ Stollstorff M. Cognitive neuroscience of attention deficit hyperactivity disorder: current status and working hypotheses. Dev Disabil Res Rev (2008) 14:261–7. 10.1002/ddrr.40 19072750 PMC4437560

[12] Frodl T Skokauskas N. Meta-analysis of structural MRI studies in children and adults with attention deficit hyperactivity disorder indicates treatment effects. Acta Psychiatr Scand (2012) 125(2):114–26. 10.1111/j.1600-0447.2011.01786.x 22118249

[13] Cortese S Castellanos FX. Neuroimaging of attention-deficit/hyperactivity disorder: current neuroscience-informed perspectives for clinicians. Curr Psychiatry Rep (2012) 14(5):568–78. 10.1007/s11920-012-0310-y 22851201 PMC3876939

[14] Nakao T Radua J Rubia K Mataix-Cols D. Gray matter volume abnormalities in ADHD: voxel-based meta-analysis exploring the effects of age and stimulant medication. Am J Psychiatry (2011) 168:11. 10.1176/appi.ajp.2011.11020281 21865529

[15] Seidman LJ Valera EM Makris N. Structural brain imaging of attention-deficit/hyperactivity disorder. Biol Psychiatry (2005) 57:1263–72. 10.1016/j.biopsych.2004.11.019 15949998

[16] Janssen J Reig S Parellada M Moreno D Graell M Fraguas D Regional gray matter volume deficits in adolescents with first episode psychosis. J Am Acad Child Adolesc Psychiatry (2008) 47:1311–20. 10.1097/CHI.0b013e318184ff48 18827723

[17] Narvaez JC Zeni CP Coelho RP Wagner F Pheula GF Ketzer CR Does comorbid bipolar disorder increase neuropsychological impairment in children and adolescents with ADHD? Rev Bras Psiquiatr (2014) 36(1):53–9. 10.1590/1516-4446-2013-1085 24604462

[18] Achenbach TM Manual for the Child Behavior Checklist/4-18 and the 1991 Profile. Burlington, VT: University of Vermont, Department of Psychiatry (1991).

[19] Althoff RR Rettew DC Faraone SV Boomsma DI Hudziak JJ. Latent class analysis shows strong heritability of the child behavior checklist-juvenile bipolar phenotype. Biol Psychiatry (2006) 60(9):903–11. 10.1016/j.biopsych.2006.02.025 16650832

[20] Hudziak JJ Althoff RR Derks EM Faraone SV Boomsma DI. Prevalence and genetic architecture of child behavior checklist-juvenile bipolar disorder. Biol Psychiatry (2005) 58:562–8. 10.1016/j.biopsych.2005.03.024 16239161

[21] Boomsma DI Rebollo I Derks EM van Beijsterveldt TC Althoff RR Rettew DC Longitudinal stability of the CBCL-juvenile bipolar phenotype: a study in Dutch twins. Biol Psychiatry (2006) 60(9):912–20. 10.1016/j.biopsych.2006.02.028 16735031

[22] Volk HE Todd RD. Does the child behavior checklist juvenile bipolar disorder phenotype identify bipolar disorder? Biol Psychiatry (2007) 62:115–20. 10.1016/j.biopsych.2006.05.036 16950211

[23] Spencer TJ Biederman J Mick E. Attention-deficit/hyperactivity disorder: diagnosis, lifespan, comorbidities and neurobiology. J Pediatr Psychol (2007) 32(6):631–42. 10.1093/jpepsy/jsm005 17556405

[24] Galanter CA Pagar DL Davies M Li W Carlson GA Abikoff H ADHD and manic symptoms: diagnostic and treatment implications. Clin Neurosci Res (2005) 5:283–94. 10.1016/j.cnr.2005.09.008

[25] Holtmann M Goth K Wöckel L Poustka F Bölte S CBCL-pediatric bipolar disorder phenotype: severe ADHD or bipolar disorder? J Neural Transm (2007) 5(2):155–61. 10.1097/CHI.0b013e3181825a68 17994189

[26] Holtmann M Bölte S Goth K Döpfner M Plück J Huss M Prevalence of the child behavior checklist-pediatric bipolar disorder phenotype in a German general population sample. Bipolar Disord (2007) 9(8):895–900. 10.1111/j.1399-5618.2007.00463.x 18076540

[27] Manning SC Miller DC Identifying ADHD subtypes using the parental and teacher rating scales of the behavior assessment scale for children. J Atten Disorder (2001) 5:41–51. 10.1177/108705470100500104

[28] Biederman J Spencer J Petty C Hyder LL O’Connor KB Surman CB Severity of the aggression/anxiety-depression/attention (A-A-A) CBCL profile discriminates between different levels of deficits in emotional regulation in youth with ADHD. Neuropsychiatr Dis Treat (2012) 8:267–76. 10.1097/DBP.0b013e3182475267 22848182 PMC3404687

[29] Spencer TJ Faraone SV Surman CB Petty C Clarke A Batchelder H Towards defining deficient emotional self regulation in youth with attention deficit hyperactivity disorder using the child behavior check list: a controlled study. Postgrad Med (2011) 123(5):50–9. 10.3810/pgm.2011.09.2459 21904086 PMC3737570

[30] Conners’ CK Conners’ Rating Scales. North Tonawanda, NY: Multi-Health Systems Inc (1990).

[31] DuPaul GJ The ADHD Rating Scale: Normative Data, Reliability and Validity. Worcester, MA: University of Massachusetts Medical Center at Worchester (1990).

[32] McCarney SB Attention Deficit Disorder Evaluation Scale – School Version. Columbia, MO: Hawthorne Educational Services (1989).

[33] Rucklidge JJ. Impacto f ADHD on the neurocognitive functioning of adolescents with bipolar disorder. Biol Psychiatry (2006) 60:921–8. 10.1016/j.biopsych.2006.03.067 16839520

[34] Henin A Mick E Biederman J Fried R Wozniak J Faraone SV Can bipolar disorder specific neuropsychological impairments in children be identified? J Consult Clin Psychol (2007) 75:210–20. 10.1037/0022-006X.75.2.210 17469879

[35] Wechsler D Padronização Brasileira. In: Figueiredo VLM, editor. Escala de Inteligência Wechsler para crianças, Terceira Edição. São Paulo: Casa do Psicólogo (2002).

[36] Brito GNO. EACI-P – Escala de avaliação do comportamento infantil para o professor. Manual. São Paulo: Ed. Entreletras (1999).

[37] Conners CK Conner’s Continuous Performance Test. Toronto, ON: Multi-Health System (2000).

[38] Santos FH Mello CB Bueno OFA. Cross-cultural differences for three visual memory tasks in Brazilian children. Percept Mot Skills (2005) 101:421–33. 10.2466/pms.101.2.421-433 16383074

[39] Meyers JE Meyers KR Rey Complex Figure Test and Recognition Trial. Professional Manual. Odessa, FL: Psychological Assessment Resource, Inc (1995).

[40] Achenbach TM Manual for the Child Behavior Checklist. Burlington, VT: University of Vermont, Department of Psychiatry (1991).

[41] Bordin IA Mari JJ Caeiro MF Validação da versão brasileira do “Child Behavior Checklist” (CBCL) (Inventário de Comportamentos da Infância e Adolescência): Dados preliminares. Revista da ABPAPAL (1995) 17:55–66.

[42] Biederman J Petty CR Day H Goldin RL Spencer T Faraone SV Severity of the aggression/anxiety-depression/attention (A-A-A) CBCL profile discriminates between different levels of deficits in emotional regulation in youth with ADHD. J Dev Behav Pediatr (2012) 33(3):236–43. 10.1097/DBP.0b013e3182475267 22278125 PMC3319866

[43] Losier BJ Mcgrath PJ Klein RM. Error patterns on the continuous performance test in non-medicated and medicated samples of children with and without ADHD: a meta-analytic review. J Child Psychol (1996) 37:971–87. 10.1111/j.1469-7610.1996.tb01494.x 9119944

[44] Riccio CA Reynolds CR Continuous performance tests are sensitive to ADHD in adults but lack specificity – a review and critique for differential diagnosis. Adult Atten Defic Disord (2001) 931:113–39. 10.1111/j.1749-6632.2001.tb05776.x 11462737

[45] Schatz AM Ballantyne AO Trauner DA. Sensitivity and specificity of a computerized test of attention in the diagnosis of attention-deficit/hyperactivity disorder. Assessment (2001) 8:357–65. 10.1177/107319110100800401 11785580

[46] Preston AS Fennell EB Bussing R. Utility of a CPT in diagnosing ADHD among a representative sample of high-risk children: a cautionary study. Child Neuropsychol (2005) 11:459–69. 10.1080/09297040591001067 16306020

[47] Homack S Riccio CA A meta-analysis of the sensitivity and specificity of the Stroop Color and Word Test with children. Arch Clin Neuropsychol (2004) 19:725–43. 10.1016/j.acn.2003.09.003 15288327

[48] Shin MS Kim YH Cho SC Kim BN Neuropsychologic characteristics of children with attention-deficit hyperactivity disorder (ADHD). Learning disorder, and tic disorder on the Rey-Osterreith Complex Figure. J Child Neurol (2003) 18:835–44. 10.1177/088307380301801203 14736077

[49] Brocki KC Bohlin G Developmental change in the relation between executive functions and symptoms of ADHD and co-occurring behaviour. Inf Child Dev (2006) 15:19–40. 10.1002/icd.413

[50] Jensen CM Steinhausen HC. Comorbid mental disorders in children and adolescents with attention-deficit/hyperactivity disorder in a large nationwide study. Atten Defic Hyperact Disord (2015) 7(1):27–38. 10.1007/s12402-014-0142-1 24942707

[51] Nee DE Wagner TD Jondes J. Interference resolution insights from a meta-analysis of neuroimaging tasks. Cogn Affect Behav Neurosci (2007) 7:1–17. 10.3758/CABN.7.1.1 17598730

[52] Whalen CK Henker B The social profile of attention-deficit hyperactivity disorder. Child Adolesc Psychiatr Clin N Am (1992) 1(2):395–410.

[53] Barkley RA Deficient emotional self-regulation: a core component of attention-deficit/hyperactivity disorder. J ADHD Relat Disord (2010) 1(2):5–37.

[54] Reebye P Elbe D. The role of pharmacotherapy in the management of self-regulation difficulties in young children. J Can Acad Child Adolesc Psychiatry (2009) 18(2):150–9. 19495438 PMC2687481

